# Ventricular Dyssynchrony Patterns in Left Bundle Branch Block, With and Without Heart Failure

**Published:** 2010-03-05

**Authors:** Hygriv B Rao, Raghu Krishnaswami, Sharada Kalavakolanu, Narasimhan Calambur

**Affiliations:** CARE Hospital, Institute of Medical Sciences, Hyderabad, India

**Keywords:** Heart Failure, Dyssynchrony, Tissue Doppler Imaging, LBBB, Normal Heart

## Abstract

**Background:**

Assessment of ventricular dyssynchrony in patients with heart failure is used for selecting candidates for cardiac resynchronization therapy (CRT). The patterns of regional distribution of dyssynchrony in a population with LBBB with and without heart failure have not been well delineated. This aspect forms the object of the study.

**Methods:**

Tissue Doppler Imaging (TDI) data of consecutive patients with heart failure and LBBB (Group A) was compared with those with LBBB and normal LV function (Group B). All patients had standard 2D-echocardigraphic examination and TDI. Tissue velocity curves obtained by placing sample volumes in opposing basal and mid segments of septal, lateral, inferior, anterior and posterior walls were analyzed. Inter ventricular dyssynchrony (IVD) was assessed by the difference between aortic and pulmonary pre ejection intervals. LV dyssynchrony (LVD) was assessed by the difference in times to peak velocity. A delay of ≥ 40 msec was considered significant for presence of IVD and LVD.

**Results:**

There were 103 patients in Group A and 25 in Group B. The mean QRS duration and PR intervals respectively were 146 ± 25 vs. 152±20 msec and 182± 47 vs. 165±36 msec. (p=NS) LVEF in the 2 groups were (32 ± 6 % vs. 61± 11%; p<  0.01). Prevalence of dyssynchrony in the HF group compared to Group B was 72% vs. 16%, (P< 0.01). Lateral wall dyssynchrony in the 2 groups was 37% vs. 0% (p< 0.01) while septal dyssynchrony was 16% vs. 16% (p- NS).

**Conclusions:**

72% of heart failure patients with LBBB have documented dyssynchrony on TDI, which has a heterogeneous regional distribution. Dyssynchrony may be seen in LBBB and normal hearts but it is does not involve the lateral wall. Septal dyssynchrony in heart failure patients may not have the same significance as lateral wall delay.

## Background

Cardiac resynchronization therapy (CRT) has proven to be beneficial in drug refractory heart failure patients for alleviation of symptoms, improving quality of life, decreasing hospitalizations and mortality [1-4]. Most of the data from multi center CRT trials has been in patients with LBBB, since it was an accepted surrogate marker of underlying ventricular dyssynchrony. Recent literature has demonstrated Tissue Doppler Imaging (TDI) to be a reliable echocardiographic tool that can profile intraventricular dyssynchrony reproducibly and has been used to help patient selection for CRT [5-8]. However the regional distribution patterns of dyssynchrony in heart failure population and in normal individuals with LBBB have not been well studied.

## Aims and Objectives

This prospective study was undertaken to assess the patterns of interventricular and left ventricular dyssynchrony (LVD) in patients with complete LBBB and heart failure. This data was compared with the dyssynchrony patterns of 25 subjects with LBBB having normal ventricular function. 

## Methods

### Patient Selection

Consecutive patients with heart failure referred for assessment of ventricular dyssynchrony by TDI and fulfilling the inclusion and exclusion criteria were studied. All patients were also subjected to a standard 2D echocardiogram prior to TDI. Individuals declared normal at annual routine physical examination but having LBBB on ECG were selected for comparison (Table 1).

### Heart Failure Patients (Group A)

#### Inclusion criteria

1. Patients aged 18 and above
2. Patients with symptomatic heart failure
3. LVEF ≤ 40% by 2 D echocardiogram
4. Complete LBBB on surface ECG

#### Exclusion Criteria

1. Predominant valvular heart disease
2. Predominant diastolic heart failure
3. Acute coronary syndromes in the last 3 months
4. Recent revascularization in the last 3 months
5. Patients with atrial fibrillation

### Normals ( Group B)

Asymptomatic consenting adults with a complete LBBB (QRS duration >  120 msec) and a normal 2D echocardiographic study were included. None of them had hypertension, coronary artery disease or pulmonary disease.

### 2 D Echocardiography

Standard echocardiography, including Doppler studies was performed with Vivid 5, GE Vingmed Ultrasound, Horten, Norway. The left ventricular end-diastolic volume, left ventricular end-systolic volume and ejection fraction were assessed by biplane Simpson's equation using the apical four- and two chamber views. Standard methods were used to assess cardiac output, Tei Index and severity of mitral regurgitation.

### TDI Methodology

Tissue Doppler imaging was performed by using apical four chamber, apical two chamber and parasternal long axis views for the long axis motion of the ventricles [9-10]. Two-dimensional echocardiography with TDI- color imaging views were optimized for pulse repetition frequency, color saturation, and sector size and depth and to allow highest possible frame rate. Pulsed-wave TDI velocities of long-axis wall motion were assessed in apical views during end-expiratory apnea, with sample volume of 5 mm positioned in the center of the analyzed segment. Care was taken to keep the incidence angle between the direction of the Doppler beam and the analyzed vector of myocardial motion as narrow as possible. The spectral Doppler signal filters were adjusted to obtain Nyquist limits between 15 and 20 cm/s with the lowest wall filter settings and the optimal gain to minimize noise. Sweep speed was set to 150 mm/s. At least three consecutive beats were stored and the images were analyzed offline using a customized software package (Echo Pac for PC, GE Vingmed Ultrasound). The echocardiography was done and analyzed by an experienced echocardiographer.

### Assessment of Dyssynchrony

**Figure 1:**
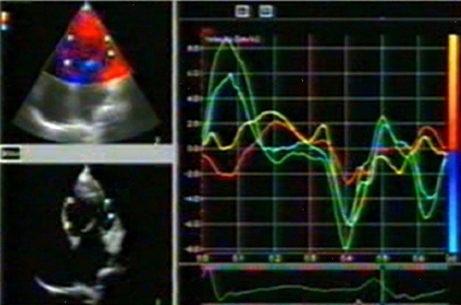
Tissue Doppler Image in a patient with Heart Failure and LBBB. Tissue Velocity curves corresponding to the sample volumes in the Lateral wall show a significant delay compared to the septal wall.

**Figure 2:**
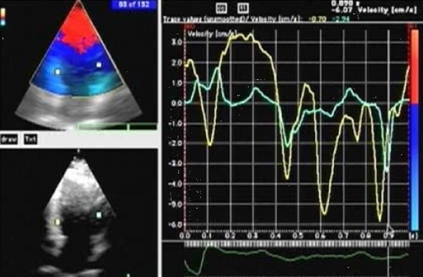
Tissue Doppler image of a normal individual with LBBB on ECG. Tissue velocity curves show a septal delay.

Interventricular dyssynchrony was evaluated by assessing the extent of interventricular mechanical delay (IVMD), defined as the time difference between left and right ventricular pre-ejection intervals. (Q to aortic flow and Q to pulmonary flow). An IVMD of > 40 msec was considered indicative of interventricular dyssynchrony. LVD was assessed from measurements of time intervals between the onset of the QRS complex and the peak regional velocity of myocardial systolic shortening, considered as a surrogate for regional electromechanical coupling intervals. Time intervals were measured in opposing basal and mid LV segments. Intraventricular (LV) dyssynchrony was determined as the difference between the longest and shortest electromechanical coupling times in the opposing basal septal, lateral, anterior, inferior and posterior segments of the LV. Any difference of 40 msec or more was considered indicative of presence intraventricular dyssynchrony. Figure 1 and 2 show examples of dyssynchrony in LBBB with and without heart failure.

### Data analysis

Statistical analysis was done by commercially available package MINITAB version 13. Continuous variables are expressed as mean + 1 standard deviation and were compared by unpaired Student t test. Discrete variables were compared using chi square test. For all comparisons, the difference between the 2 groups was considered significant at a level of 5%.

## Results

**Figure 3:**
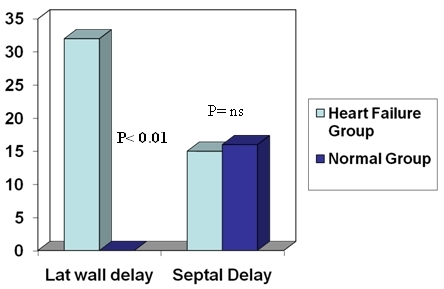
Lateral wall delay was found only in the heart failure group while the prevalence of septal delay was similar in Heart Failure group and controls.

Of the 233 heart failure patients referred for TDI 103 had complete LBBB and formed the cohort for this study. The baseline characteristics of the 103 patients and 25 controls with LBBB are summarized in Table 1. Interventricular delay in the form of LV delay was demonstrated in 42 patients (41 %) in Group A and in 3 (12%) in Group B. 74 of the patients (72%) demonstrated intraventricular dyssynchrony in 112 regions. 36 of these patients (35%) had delay in 1 region, 30 (29%) had delay in 2 regions and 7 (7%) had delay in 3 regions. Overall 38 patients (37%) had lateral wall delay, and 16 (16%) had septal delay. In patients with a single region of delay, the occurrence of lateral wall delay was the commonest (17 patients, 47%), followed by delay at septal (9 patients, 36%), posterior (7 patients, 11%), and inferior wall (3 patients, 6%). In the 31 patients with 2 regions of delay, lateral wall was involved in 15, in association with inferior (9) posterior (4), or anterior wall (2); septal wall was involved in 6 patients, in association with inferior (5) or anterior (1); and posterior wall involvement in association with inferior wall was seen in 9 patients. The 7 patients with 3 regions of dyssynchrony had posterior and inferior wall involvement in association with either lateral wall (6) or septal (1). In patients who had LVD the mean delay was 72+/- 30 msec. In comparison 4 patients (16 %) in Group B had ventricular dyssynchrony (P < 0.01) all of them had septal delay. The prevalence of septal delay in the 2 groups was similar (18% vs. 16%, p = ns) (Figure 3).

## Discussion

Prevalence of dyssynchrony in heart failure patients varies depending on methodology and cut off values used. In our study we measured LVD from onset of QRS to peak of respective tissue velocity curve and used a cutoff value of 40 msec. This value was obtained by assessment of 20 normal individuals in our lab and was greater than 2 SD of LVD. This study documented LVD in 72% of heart failure patients with LBBB, thus showing a lack of consistent correlation between presence of dyssynchrony assessed by TDI and complete LBBB and reinforces the observation that a wide QRS does not always predict dyssynchrony  [11-13]. An important observation in this study is the heterogeneous distribution pattern of dyssynchrony in the heart failure population. Only one in three patients had dyssynchrony in the lateral wall, and slightly over half had dyssynchrony in areas other than lateral wall. Further, 37 patients had dyssynchrony in multiple areas (2 or more) suggesting that these represent the presence of more extensive dyssynchrony. The reason for conflicting results in recent publications attempting to correlate dyssynchrony with benefit from CRT, may stem from variable patterns of dyssynchrony proving to be more important than mere presence of dyssynchrony [14,15]. The benefit of CRT in a patient population where there is a lack of concordance between the pacing site and the site of delay is uncertain and needs to be assessed on a larger scale. The documentation of dyssynchrony in individuals with LBBB and otherwise normal hearts raises the issue of specificity of this parameter in assessment of heart failure patients. The mean severity of dyssynchrony was no different from heart failure population showing that raising the cut off value would not help differentiate these two groups. On the other hand comparison of regional distribution patterns revealed that the normal group had dyssynchrony only in the septal wall. Surprisingly the prevalence of septal wall delay was similar in both the groups. This makes the significance of isolated septal delay in heart failure patients unclear with respect to implantation of CRT. It is known that all people with LBBB do not develop heart failure as also all right ventricular paced patients. To answer the question if a subset of patients with LBBB and dyssynchrony are more likely to develop heart failure in the future, a long term follow up study will be required. The newer technologies of echocardiographic strain and strain rate imaging enable a reliable and comprehensive assessment of myocardial function.  Two-dimensional strain imaging by speckle tracking and TDI-derived strain imaging are well suited to detecting and defining LV dyssynchrony and they have already proved to be useful for both the selection of patients who might benefit from CRT [16-18]. The predictive value for LV functional response to CRT has improved with the use of combination of parameters of systolic dyssynchrony based on TDI longitudinal velocity and parameters of systolic dyssynchrony based on radial strain data obtained by speckle tracking (2D-strain imaging) [17].

## Conclusions

LV dyssynchrony is present in 72% of heart failure population with complete LBBB. The regional distribution patterns of dyssynchrony are heterogenous. Lateral wall delay is seen only in heart failure patients while septal delay is present similarly in heart failure patients and normal individuals with LBBB. Prospective follow up of this population may result in correlation of dyssynchrony patterns and development of LV dysfunction.

## Figures and Tables

**Table 1:**
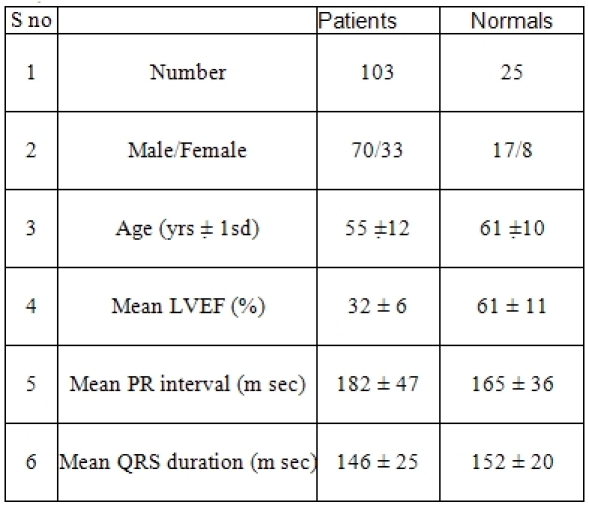
Baseline Characteristics
